# Effect of intensive inpatient physical therapy on whole-body indefinite symptoms in patients with whiplash-associated disorders

**DOI:** 10.1186/s12891-019-2621-1

**Published:** 2019-06-05

**Authors:** Takayoshi Matsui, Makoto Iwata, Yuzo Endo, Nobuyuki Shitara, Shuntaro Hojo, Hideoki Fukuoka, Kazuhiro Hara, Hiroshi Kawaguchi

**Affiliations:** 1 Head of Orthopaedics and Spine Department, Tokyo Neurological Center, Toranomon 4-1-17, Minato-ku, Tokyo, 105-0001 Japan; 2 Department of Neurosurgery, Matsui Hospital, Kan-nonji 739, Kagawa, 768-0013 Japan

**Keywords:** Whiplash injuries, Neck muscles, Therapy, Electric stimulation

## Abstract

**Background:**

A considerable number of patients with whiplash-associated disorders (WAD) report variable and indefinite symptoms involving the whole body, despite there being no evidence of direct injuries to organs other than the neck. However, little is known about their management or underlying mechanism. This study examined the effect of intensive physical therapy at the cervical muscles in patients with WAD reporting whole-body indefinite symptoms.

**Methods:**

A total of 194 hospitalized patients with WAD who were resistant to outpatient care by reporting whole-body indefinite symptoms between May 2006 and May 2017 were enrolled in this observational study. All patients underwent daily physical therapies by low-frequency electric stimulation therapy and far-infrared irradiation to the cervical muscles during hospitalization. Self-rated records in the medical interview sheets on 22 representative whole-body symptoms at admission and discharge were compared.

**Results:**

The number of symptoms was markedly decreased by the physical therapies during hospitalization. Almost all symptoms showed recovery rates of more than 80% at discharge as compared to those at admission. Although the percentage of patients reporting at least four of the 22 representative indefinite symptoms was 99.0% at admission, it decreased to 7.7% at discharge. Sixteen percent of patients recovered completely without any residual symptoms. The mean number of symptoms significantly decreased from 13.1 at admission to 2.0 at discharge. Notably, symptoms other than those in the neck or shoulder recovered to a greater extent than those in the neck or shoulder.

**Conclusions:**

This study, for the first time, examined the management of whole-body indefinite symptoms in patients with WAD. The intensive physical therapy markedly improved the symptoms, suggesting the involvement of cervical muscles in the pathogenesis.

**Trial registration:**

UMIN000035435 (Retrospectively registered on Jan 3, 2019).

**Electronic supplementary material:**

The online version of this article (10.1186/s12891-019-2621-1) contains supplementary material, which is available to authorized users.

## Background

Whiplash neck injury, which commonly occurs in motor vehicle accidents, causes acute and chronic whiplash-associated disorders (WAD) [1–3]. Its cumulative annual incidence is reported to be more than 300/100,000 people [4]. The Quebec Task Force defined whiplash neck injury as an injury caused by an acceleration-deceleration mechanism of energy transfer to the neck [5]. The energy may impact and cause injury to various components of the neck including bones, intervertebral discs, facet joints, spinal cord, nerve roots, and cervical muscles [5–7]. Mostly, however, the mechanical stress on the neck from motor vehicle accidents is not so strong or severe enough to injure bones, intervertebral discs, facet joints, spinal cord, or nerve roots, although it may cause functional disorders like cramps or spasms of cervical muscles [4, 8]. Hence, pain or stiffness of the neck or shoulder, the local and main symptom caused by nociceptive mechanical stimulus of the muscles, usually recovers within two to three weeks following the injury by conventional outpatient care, such as medication or neck rest [1, 8]. Alternatively, up to 30–50% of patients with WAD report variable and indefinite symptoms involving the whole body, despite there being no evidence of direct injury to organs other than the neck [1–4, 9, 10]. The reported whole-body indefinite symptoms include headache, vertigo or dizziness, palpitation, chest tightness, vision loss, dazzling, dry eyes, dry mouth, nausea or appetite loss, gastrointestinal symptoms (stomachache, diarrhea, and constipation), hyperhidrosis, cold sensation or poor circulation, unstable blood pressure, unknown fever, sleeping disorder, general malaise or fatigue, depression, distraction or obsession, irritability, and lack of endurance.

Treatment for patients with WAD remains controversial. A review reports that some active treatments, although not specified, have a tendency to be more effective than only resting of the neck [11]. Several reports have shown that pharmacological interventions such as paracetamol, nonsteroidal anti-inflammatory drugs (NSAIDs), and opioids have slight or moderate effects [12, 13]. However, these studies are limited to the management of local pain in the neck or shoulder, performed as outpatient care for acute WAD. The management of other whole-body indefinite symptoms or intensive treatment by hospitalization has not been extensively examined.

We have previously reported that cervical muscle disorders may possibly be associated with these indefinite and variable symptoms and propose a new medical concept, namely cervical neuro-muscular syndrome [14]. On the basis of our clinical experience, we believe that functional disorders in cervical muscles, such as cramps or spasms after injury, are responsible not only for neck and shoulder symptoms but also for other whole-body symptoms of WAD. On palpation of 34 points of the neck, which is the original diagnostic method used at our institutions (Additional file 2: Figure S1), we observed that patients with WAD exhibit tenderness and hardening at specific sites of the posterior and lateral cervical muscles, such as trapezius, semispinalis capitis, splenius capitis, and sternocleidomastoid. These muscle lesions cannot be diagnosed using images such as plain radiographs or magnetic resonance imaging (MRI). For the treatment, we attempted prescribing medication or neck rest with a cervical collar for functional disorders of the cervical muscles as outpatient care. Although these were partly effective for local symptoms of the neck or shoulder, they were hardly effective for other symptoms in the whole body.

Among the many interventions for local modulation of the cervical muscles, low-frequency electric stimulation has recently been reported to be effective for recovery of muscle tone of the erector spinae [15, 16]. In addition, a randomized, double-blind, placebo-controlled pilot study has shown that far-infrared irradiation significantly decreased the stiffness of cervical muscles [17]. We also believe that a combination of the two physical therapies can decrease tenderness and hardening of the cervical muscles much more effectively than conventional treatments, through the aforementioned palpation of the neck. The present study therefore examined the effect of a combination of the two physical therapies at the cervical muscles of inpatients with WAD who were resistant to outpatient care and showed variable symptoms in the whole body.

## Methods

### Study design

This study is an observational study.

#### Patients

Among patients who visited our institutions with whiplash neck injuries due to motor vehicle accidents between May 2006 and May 2017, a considerable number of patients reported one or more indefinite symptoms in the whole body besides neck or shoulder symptoms. All underwent conventional outpatient care, such as medication and neck rest using a cervical collar. Outpatient care does not include physical therapies at the cervical muscles. This is because the outpatient facilities at our institutions, like that at most of hospitals in Japan, do not have equipment for physical therapies, whereas inpatient facilities do.

There was no definite criterion for hospitalization of the patients. In principle, hospitalization was decided through consensus between the patients and physicians, independently of the severity or frequency of the symptoms and the presence of neurological disorder or psychological distress. The main reason for hospitalization is resistance to outpatient care because of persistent indefinite symptoms involving the whole body that need not only more intensive treatments but also detailed examination in other organs. Patients with a history of any of the aforementioned symptoms before the motor vehicle accident were excluded. To avoid the effects of musculoskeletal and neuromuscular dysfunctions due to congenital or degenerative disorders apart from whiplash injury, those with cervical spondylosis, disk degeneration or herniation, ligament ossification, spinal deformity or scoliosis on plain radiographs and MRI, and those with neurologic deficits in the extremities on examination at admission, were also excluded. Self-rated medical interview sheets to evaluate the whole-body symptoms (Additional file 2: Figure S2) were collected at admission and discharge.

In total, 211 patients who were resistant to outpatient care for 14 to 61 days (27.2 days on average) and hospitalized at our institutions were enrolled in this observational study. Among these patients, 17 were excluded after enrollment: six patients were diagnosed with specific diseases in other organs after admission, four patients received pharmacological or non-pharmacological treatments in addition to physical therapies at their insistence, four patients discharged themselves from the hospital for unknown reasons, two patients quit physical therapy because of skin disorders that were judged as therapy-related by physicians, and one patient refused to leave the hospital independent of the presence of symptoms even after 90 days. After excluding these patients, 194 patients, with a mean age of 45.6 years, including 82 men (45.4 years) and 112 women (45.7 years) met the aforementioned criteria (Table 1). Time after the whiplash injury at admission was 14 to 89 days (36.6 days on average). The period of hospitalization from admission to discharge was 11 to 88 days (46.1 days on average). None of the patients underwent re-hospitalization because of aggravation of the symptoms until their last visit to our institutions (0 to 54 days; 18.3 days on average, after discharge).

**Table 1:** Patient backgrounds

Age	Gender	Number (percentage) of patients
10–19	Male	0 (0)
	Female	4 (2.1)
20–29	Male	7 (3.6)
	Female	14 (7.2)
30–39	Male	26 (13.4)
	Female	25 (12.9)
40–49	Male	19 (9.8)
	Female	27 (13.9)
50–59	Male	13 (6.7)
	Female	18 (9.3)
60–69	Male	11 (5.7)
	Female	13 (6.7)
70–79	Male	6 (3.1)
	Female	9 (4.6)
80–89	Male	0 (0)
	Female	2 (1.0)
Total		194 (100.0)

#### Intervention

**Fig. 1:**
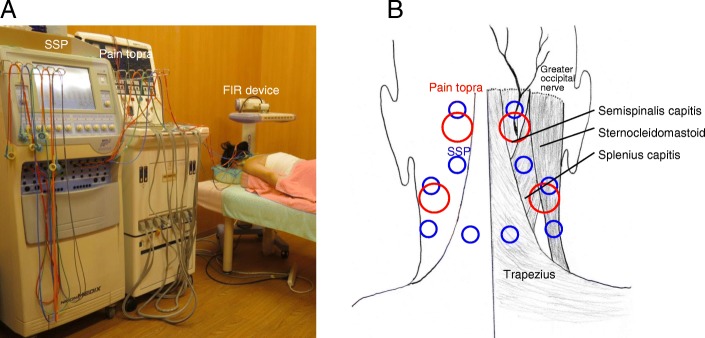
**a** A scene photograph of treatment with low-frequency electric stimulation with silver spike point (SSP) and pain topra, combined with far-infrared irradiation (FIR). **b** A representative example of SSP (blue circle) and pain topra (red circle) stimulation sites (ten and two, respectively). As most patients report headache and pain or stiffness in the neck and shoulder, both stimulations are performed at the semispinalis capitis muscle with the greater occipital nerve (CP sites in Additional file 2: Figure S1) and the trapezius and splenius capitis muscles (C and P sites, respectively, in Additional file 2: Figure S1)

During hospitalization, the patients underwent physical therapies: low-frequency electric stimulation therapy and far-infrared irradiation at the cervical muscles for 15 min twice daily (Fig. 1A). None of the other treatments including medication, injection, external fixation, or cervical traction were administered. A combination of silver spike point (SSP; Nihon Medix, Chiba, Japan) and pain topra (LCF-30; Celcom, Inc., Fukuoka, Japan) were used for low-frequency electrical therapy. Ten and four sites were stimulated by SSP and pain topra, respectively (Fig. 1B). CERAPIA 3300 (Nihon Medix, Chiba, Japan) was used for far-infrared ray irradiation to the entire neck.

Sites of low-frequency electric stimulations were determined on the basis of hardness and tenderness of 34 points of the neck upon palpation by eight physicians at our institutions (Additional file 2: Figure S1). Although neither the inter-rater nor intra-rater reliability of our palpation technique was tested, this original technique has meticulously been unified and made consistent among physicians according to the strict and frequent orientation by Dr. Matsui, who is the pioneer of this method [14]. For example, the semispinalis capitis muscle with the greater occipital nerve (CP sites in Additional file 2: Figure S1) was stimulated in patients with headache. The trapezius and splenius capitis muscles (C and P sites, respectively, in Additional file 2: Figure S1) were stimulated in patients with pain or stiffness of the neck and shoulder (Fig. 1b).

#### Statistical analysis

Statistical analyses were performed using SPSS 16.0 J for Windows. A *P* value less than 0.05 was considered to be statistically significant; all reported *P* values were two-sided. As the sample size (*n* = 194) is large enough (> 30 or 40), the central limit theorem can be applied to clarify that the data are normally distributed and violation of the normality assumption should not cause major problems [18]. Hence, the paired Student’s t-test was used to examine the difference in the number of symptoms between admission and discharge. The difference in the number of patients with each symptom between admission and discharge was evaluated using the chi-square test, whereas Fisher’s exact test was used for symptoms for which the number of patients was five or fewer at discharge.

## Results

The number of patients with the 22 representative symptoms involving the whole body at admission and discharge is shown in Table 2. At admission, more than 70% of patients reported headache, cold sensation or poor circulation, sleeping disorders, and general malaise or fatigue, apart from the local symptoms in the neck or shoulder. More than 50% reported vertigo or dizziness, vision loss, nausea or appetite loss, gastrointestinal symptoms (stomachache, diarrhea, or constipation), hyperhidrosis, depression, distraction or obsession, irritability, and lack of endurance. In addition, more than 30% of patients reported palpitation, chest tightness, dazzling, dry eyes, and dry mouth. At discharge, however, all indefinite symptoms except for sleeping disorder showed recovery rates higher than 80%. Headache, vertigo or dizziness, dry mouth, gastrointestinal symptoms (stomachache, diarrhea or constipation), hyperhidrosis, and sleeping disorder were significantly improved at discharge (*P <* 0.05). Notably, all patients (100%) with depression, distraction or obsession at admission recovered at discharge. More interestingly, pain or stiffness in the neck or shoulder, which are thought to be the main symptoms of WAD, showed the lowest recovery rate (50–60%).

**Table 2:** The numbers (percentages) of patients with the representative 22 symptoms at admission and discharge, and recovery rates

Symptom	Number (percentage) of patientsat admission (Total = 194)	Number (percentage) of patients at discharge (Total = 194)	Recovery rate	*P*-value
Headache	170 (87.6)	31 (16.0)	81.8	0.022
Neck pain or stiffness	186 (95.9)	85 (43.8)	54.3	0.273
Shoulder pain or stiffness	185 (95.4)	76 (39.2)	58.9	0.077
Vertigo or dizziness	123 (63.4)	17 (8.8)	86.2	0.006
Palpitation	81 (41.8)	4 (2.1)	95.1	0.198
Chest tightness	63 (34.5)	7 (3.6)	89.6	0.188
Vision loss	114 (58.8)	5 (2.6)	95.6	0.664
Dazzling	95 (49.0)	4 (2.1)	95.8	0.056
Dry eyes	88 (45.4)	5 (2.6)	94.3	0.132
Dry mouth	71 (36.6)	7 (3.6)	90.1	0.010
Nausea or appetite loss	107 (55.2)	3 (1.5)	97.2	0.577
Stomachache, diarrhea, or constipation	115 (59.3)	17 (8.8)	85.2	0.011
Hyperhidrosis	104 (53.6)	17 (8.8)	83.7	< 0.0001
Cold sensation or poor circulation	141 (72.7)	26 (13.4)	81.6	0.052
Unstable blood pressure	45 (23.2)	3 (1.5)	93.3	0.135
Unknown fever	48 (24.7)	5 (2.6)	89.6	0.237
Sleeping disorder	147 (75.8)	37 (19.1)	74.8	0.011
General malaise or fatigue	175 (90.2)	18 (9.3)	89.7	0.840
Depression	126 (64.9)	0 (0)	100.0	–
Distraction or obsession	99 (51.0)	0 (0)	100.0	–
Irritability	119 (61.3)	1 (0.5)	99.2	0.613
Lack of endurance	126 (64.9)	5 (2.6)	96.0	0.112

The *P*-value for difference in symptoms between admission and discharge was evaluated using the chi-square test, while Fisher’s exact test was used when the number of patients was ≤5 at discharge

**Fig. 2:**
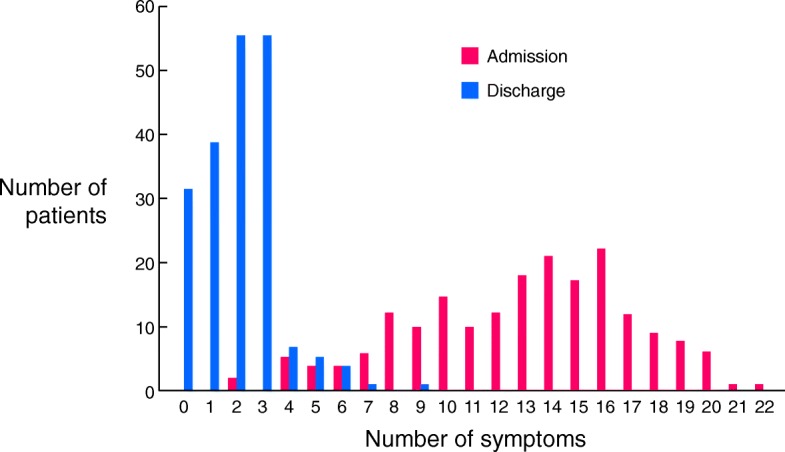
Numbers of patients with their respective number of symptoms at admission (red bars) and discharge (blue bars)

The number of patients and their respective numbers of symptoms at admission and discharge are shown in Fig. 2, and they reveal a remarkable decrease in the number of symptoms during the hospitalization. Overall, 99.0% of the patients reported at least 4 symptoms at admission; however, this decreased to 7.7% at discharge (Table 3). Sixteen percent of patients completely recovered without any remaining symptoms. The number of symptoms present at admission were significantly decreased compared to that at discharge (13.1 ± 4.1 [mean ± standard deviation] versus 2.0 ± 1.4; *P <* 0.0001).

**Table 3:** Proportion of patients with their respective number of symptoms at admission and discharge

Number of symptoms	0	1	2	3	≥4	Total
Number (percentage) of patients at admission	0	0	2 (1.0)	0 (0)	192 (99.0)	194 (100.0)
Number (percentage) of patients at discharge	31 (16.0)	38 (19.6)	55 (28.4)	55 (28.4)	15 (7.7)	15 (7.7)

## Discussion

This study, for the first time, examined the management of whole-body indefinite symptoms in patients with WAD, and found that the intensive physical therapy at the cervical muscles markedly improved the symptoms. With regard to the underlying mechanism, possible involvement of the autonomic nervous system has been proposed [19]. Autonomic nervous system dysfunction has recently been reported to play a role in painful musculoskeletal conditions such as chronic low back pain [20], fibromyalgia [21], and brachialgia [22]. The autonomic nervous system, which is responsible for regulating unconscious actions, consists of parasympathetic and sympathetic nervous systems. Because of its anatomical location, the parasympathetic system is referred to as having a craniosacral outflow, whereas the sympathetic nervous system has a thoracolumbar outflow. The parasympathetic system predominates during rest conditions, controlling miosis, lacrimation, salivation, pulsation, sweating, digestion, defecation, and peripheral circulation. Hence, the whole-body symptoms in WAD might possibly be related to dysfunction of the parasympathetic nerve that passes through cervical muscles. We assume that physical compression of the parasympathetic nerve caused by cervical muscle cramps or spasms may lead to the whole-body symptoms. However, considering that pain or stiffness in the neck or shoulder showed the lowest recovery rate by the physical therapies (Table 2), other mechanisms independent of the modulation of cervical muscles cannot be denied. In fact, the physical therapies used in this study: low-frequency electric stimulation and far-infrared irradiation, are reported to modulate not only muscle tone and stiffness [15–17] but also nerve regeneration and repair directly [23–25]. Therefore, a major limitation of this study is the lack of a detailed evaluation of muscle tone and stiffness before and after the intervention. Although we believe that the tenderness and hardening of cervical muscles significantly improved during hospitalization on palpation of the neck, a routine diagnostic method at our institutions (Additional file 2: Figure S1), objective or more reliable subjective tools are needed to examine the severity of cervical muscle functional disorders. For example, ultrasound elastography, which has been used in oncology to detect malignant lesions in soft tissues, is now applied to measure the mechanical properties of muscle in patients with multiple sclerosis spasticity [26]. We are now performing a prospective study in patients with various cervical disorders to measure the hardness and stiffness of cervical muscles using shear-wave elastography, a representative ultrasound elastography technique [27].

Another major limitation of the present study is that this is an observational study. In other words, this study lacks a control group that did not undergo physical therapies during hospitalization. Although the present inpatient population did not receive any other active treatments, the possibility that various symptoms improved merely by resting during hospitalization cannot be denied. Initially, this project was planned as a prospective randomized controlled trial; however, neither the authorities of the Japanese Ministry of Health, Labour and Welfare (presently called Pharmaceuticals and Medical Devices Agency) nor the IRB gave us permission to set up a control group because of ethical problems; only resting without any active intervention for hospitalized patients was not allowed. However, the inpatients enrolled in this study had already undergone conventional outpatient care, including neck rest with a cervical collar except local physical therapies for 14 to 61 days (27.2 days on average) before the hospitalization, but were resistant to care. As shown in Additional file 1: Table S1, which shows a comparison of the proportion of patients with the 22 representative symptoms between the first visit (*n* = 139) and admission (*n* = 194), the percentages of patients with symptoms other than those in the neck or shoulder did not decrease but increased during outpatient care, including resting neck rest. Therefore, it seems improbable that all symptoms recovered to such a drastic extent only by resting, even in the hospital.

There are many confounding factors to be considered in this study. Especially, psychological distress is a common problem following a whiplash injury and may play a key role in chronicity of the symptoms and even the recovery rate [28, 29]. Actually, in this study, more than a half of the patients reported psychological symptoms such as sleeping disorder, general malaise or fatigue, depression, distraction or obsession, irritability, and lack of endurance at admission (Table 2). Although these psychological symptoms showed recovery rates more than 70% at discharge, these cannot be explained only by parasympathetic system dysfunction. Meanwhile, it is known that a significant proportion of patients with WAD are also diagnosed with post-traumatic stress disorder (PTSD) and patients with PTSD show autonomic nervous system dysfunction [30]. Further, it is true that many physicians have a perception that psychological aspects associated with compensation may be linked to whole-body indefinite symptoms. In contrast, these symptoms might be secondary to various and indefinite disorders. Therefore, whether these psychological symptoms are causes or consequences of indefinite symptoms of WAD is still controversial. Evaluation of the psychological conditions of patients in more detail will be needed as a routine practice.

The economic impact of medical interventions should be considered with increasing health care expenditures and limited resources. The inpatient physical therapy performed in this study, twice daily for an average of 46 days, is a costly treatment for both health care providers and individuals. Costs associated with WAD are estimated to be approximately US $4 billion per year in the UK, which are attributed to the health service as well as the long term disability of the patients [31]. Although a recent review of the literature failed to find any cost-effectiveness evaluations of treatments for WAD [32], feasibility of the present costly treatment as a therapeutic choice may be questionable. Development of more simple and feasible treatments that can be performed even in outpatient care or at home, such as a portable device for the physical therapy, will be the next task ahead of us.

## Conclusions

This study for the first time examined the management of whole-body indefinite symptoms in patients with WAD. Intensive physical therapies at the cervical muscles during hospitalization markedly improved these symptoms. Almost all the 22 representative symptoms showed recovery rates of greater than 80%. Although most patients reported at least four symptoms at admission, these decreased to less than 10%. Moreover, approximately one-sixth of the patients showed no symptoms at discharge. The mean number of symptoms was significantly decreased from 13.1 at admission to 2.0 at discharge. Interestingly, symptoms other than those in the neck or shoulder recovered to a greater extent than did those in the neck or shoulder. This suggests that the whole-body indefinite symptoms in WAD may be derived from local disorders in the cervical muscles, possibly via parasympathetic nerve dysfunction.

## Additional files

Additional file 1: **Table S1.** Proportion of patients with the 22 representative symptoms at the first visit and admission of the present study population. Self-rated medical interview sheets at the first visit were retrospectively collected, so that the interview sheets of 55 patients were missing (total number = 139 at the first visit versus 194 at admission). Please compare the percentages in parentheses, but not the numbers. (DOC 64 kb)

Additional file 2: **Figure S1.** Thirty-four points of the neck to determine muscle lesions on palpation in patients with WAD. **Figure S2.** Self-rated medical interview sheet to evaluate the whole-body symptoms. (PPT 283 kb)

## Abbreviations

FIR: Far-infrared irradiation
IRB: Institutional review board
MRI: Magnetic resonance imaging
NSAIDs: Nonsteroidal anti-inflammatory drugs
SSP: Silver spike point
WAD: Whiplash-associated disorders
