# Supplementary material for: Effect of intensive inpatient physical therapy on whole-body indefinite symptoms in patients with whiplash-associated disorders
Source: BMC Musculoskelet Disord. 2019 Jun 5;20:251. doi: 10.1186/s12891-019-2621-1 (PMC6549292; doi:10.1186/s12891-019-2621-1)
Supplement: Supplementary file 1 — Table S1. Proportion of patients with the 22 representative symptoms at the first visit and admission of the present study population. Self-rated medical interview sheets at the first visit were retrospectively collected, so that the interview sheets of 55 patients were missing (total number = 139 at the first visit versus 194 at admission). Please compare the percentages in parentheses, but not the numbers. (DOC 64 kb) [file 12891_2019_2621_MOESM1_ESM.doc]

**Supplementary Table 1.** Proportion of patients with the representative 22 symptoms at the first visit and admission of the present study population.

Self-rated medical interview sheets at the first visit were retrospectively collected, so that the interview sheets of 55 patients were missing (total number = 139 at the first visit versus 194 at admission). Please compare the percentages in parentheses, but not the numbers.

| **Symptom** | **Number (percentage)**  **of patients**  **at the first visit**  **(Total=139)** | | **Number (percentage)**  **of patients**  **at admission**  **(Total=194)** | | |
| --- | --- | --- | --- | --- | --- |
| Headache | 106 (76.3) |  |  | 170 (87.6) |  |
| Neck pain or stiffness | 138 (99.3) |  |  | 186 (95.9) |  |
| Shoulder pain or stiffness | 135 (97.1) |  |  | 185 (95.4) |  |
| Vertigo or dizziness | 57 (41.0) |  |  | 123 (63.4) |  |
| Palpitation | 34 (24.4) |  |  | 81 (41.8) |  |
| Chest tightness | 36 (25.9) |  |  | 67 (34.5) |  |
| Vision loss | 36 (25.9) |  |  | 114 (58.8) |  |
| Dazzling | 45 (32.4) |  |  | 95 (49.0) |  |
| Dry eyes | 31 (24.5) |  |  | 88 (45.4) |  |
| Dry mouth | 36 (25.9) |  |  | 71 (36.6) |  |
| Nausea or appetite loss | 69 (49.6) |  |  | 107 (55.2) |  |
| Stomachache, diarrhea, or constipation | 45 (32.3) |  |  | 115 (59.3) |  |
| Hyperhidrosis | 52 (37.4) |  |  | 104 (53.6) |  |
| Cold sense or poor circulation | 38 (27.3) |  |  | 141 (72.7) |  |
| Unstable blood pressure | 39 (28.1) |  |  | 45 (23.2) |  |
| Unknown fever | 29 (20.9) |  |  | 48 (24.7) |  |
| Sleeping disorder | 91 (65.5) |  |  | 147 (75.8) |  |
| General malaise or fatigue | 115 (82.7) |  |  | 175 (90.2) |  |
| Depression | 71 (51.1) |  |  | 126 (64.9) |  |
| Distraction or obsession | 48 (34.5) |  |  | 99 (51.0) |  |
| Irritability | 46 (33.1) |  |  | 119 (61.3) |  |
| Lack of endurance | 43 (31.9) |  |  | 126 (64.9) |  |
