# Supplementary material for: Effect of intensive inpatient physical therapy on whole-body indefinite symptoms in patients with whiplash-associated disorders
Source: BMC Musculoskelet Disord. 2019 Jun 5;20:251. doi: 10.1186/s12891-019-2621-1 (PMC6549292; doi:10.1186/s12891-019-2621-1)
Supplement: Supplementary file 2 — Figure S1. Thirty-four points of the neck to determine muscle lesions on palpation in patients with WAD. Figure S2. Self-rated medical interview sheet to evaluate the whole-body symptoms. (PPT 283 kb) [file 12891_2019_2621_MOESM2_ESM.ppt]

## Slide 1
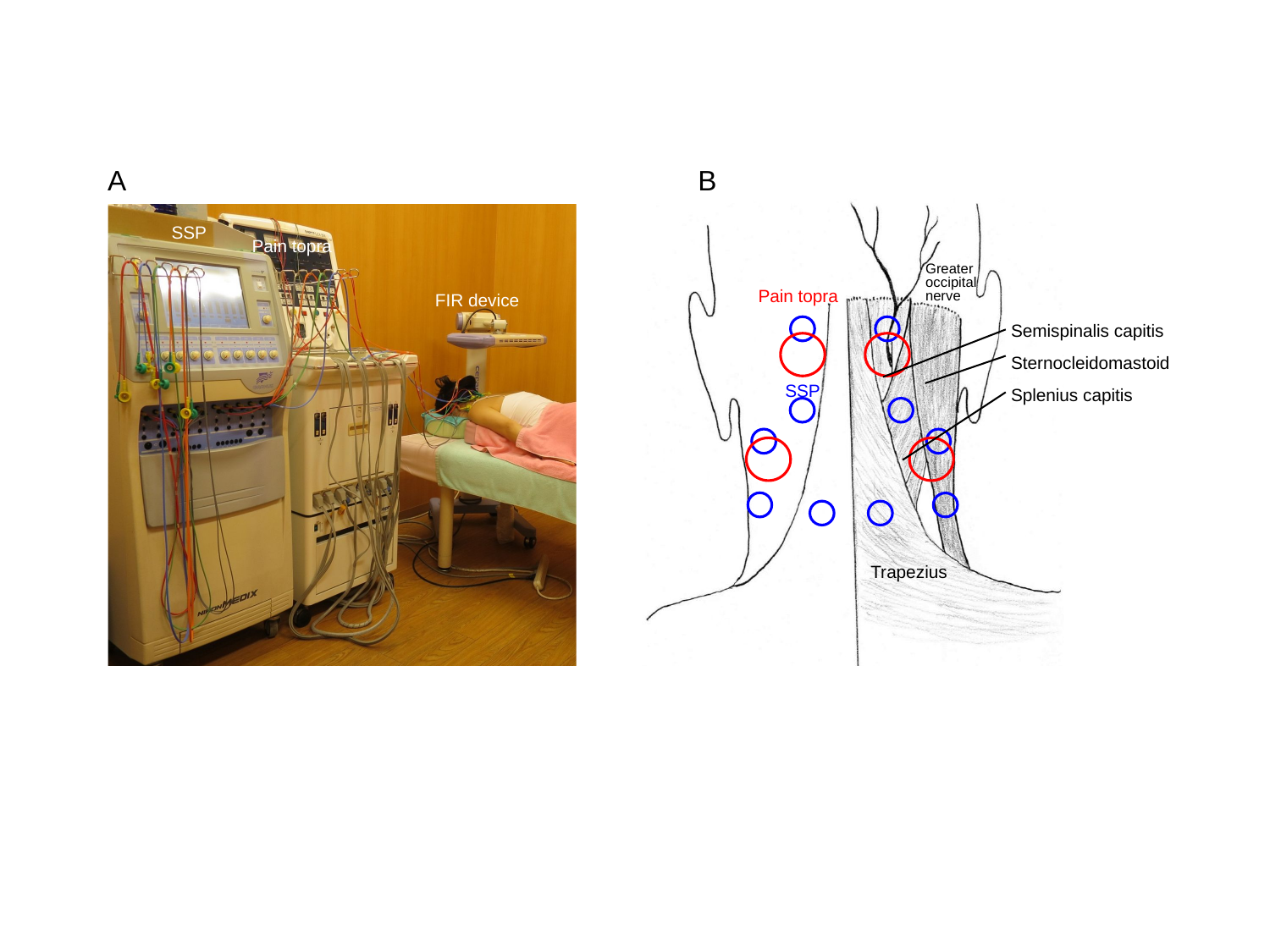

A
B
SSP
Pain topra
Greater
occipital
nerve
Pain topra
FIR device
Semispinalis capitis
Sternocleidomastoid
SSP
Splenius capitis
Trapezius

## Slide 2
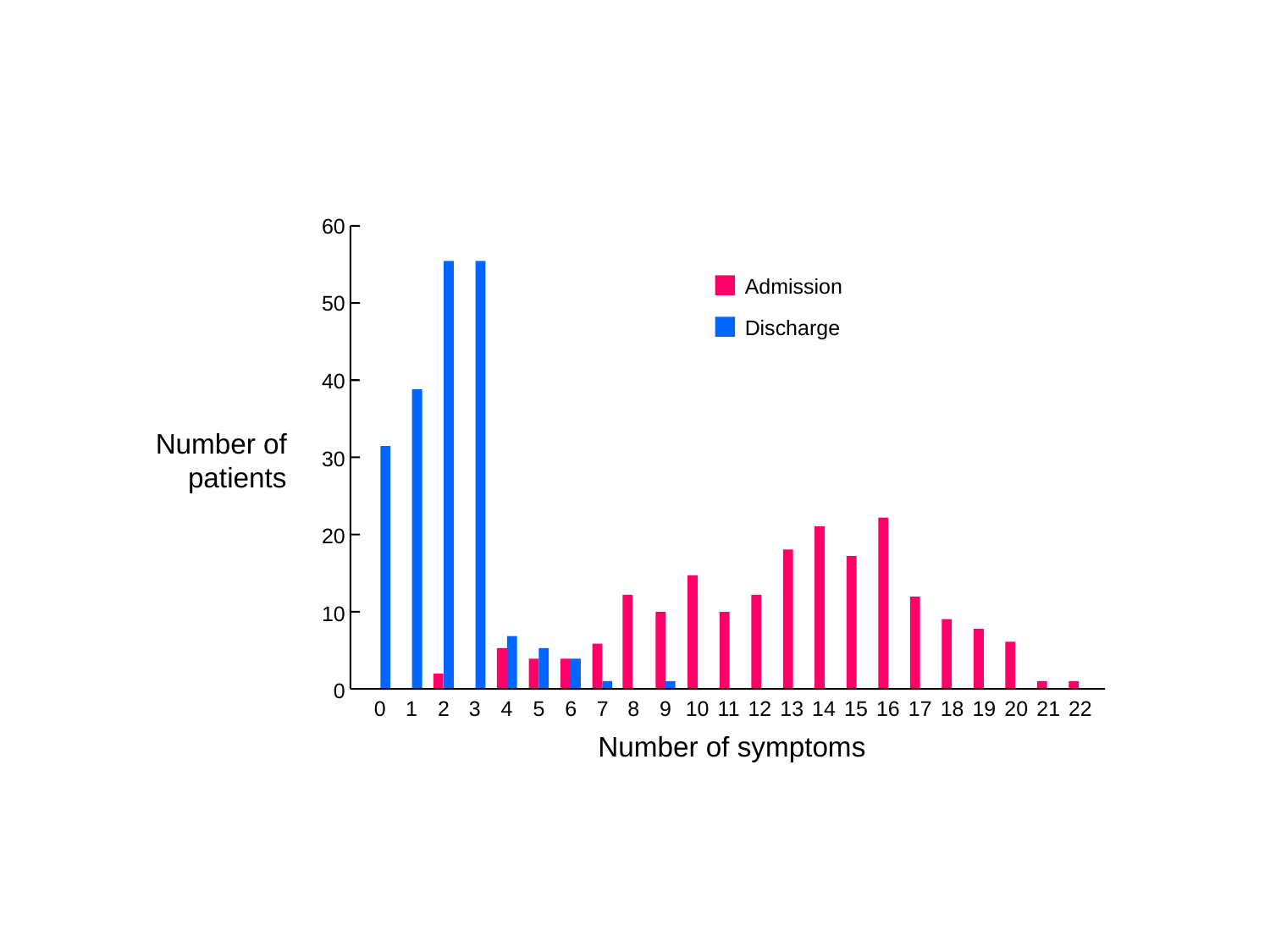

60
Admission
50
Discharge
40
Number of
patients
30
20
10
0
0
1
2
3
4
5
6
7
8
9
10
11
12
13
14
15
16
17
18
19
20
21
22
Number of symptoms
